# Germline competent mesoderm: the substrate for vertebrate germline and somatic stem cells?

**DOI:** 10.1242/bio.058890

**Published:** 2021-10-14

**Authors:** Aaron M. Savage, Ramiro Alberio, Andrew D. Johnson

**Affiliations:** 1 School of Pharmacy, Division of Stem Cell and Regenerative Medicine, University of Nottingham, University Park, Nottingham, NG7 2RD, UK; 2 School of Biosciences, Stem Cell Biology, Reprogramming and Pluripotency, University of Nottingham, Sutton Bonington Campus, Loughborough, LE12 5RD, UK; 3 School of Life Sciences, Division of Cells, Organisms and Molecular Genetics, University of Nottingham, Medical School, Queen's Medical Centre, Nottingham, NG7 2UH, UK

**Keywords:** Primordial germ cells, Haematopoietic stem cells, Development, Stem cells

## Abstract

*In vitro* production of tissue-specific stem cells [e.g. haematopoietic stem cells (HSCs)] is a key goal of regenerative medicine. However, recent efforts to produce fully functional tissue-specific stem cells have fallen short. One possible cause of shortcomings may be that model organisms used to characterize basic vertebrate embryology (*Xenopus*, zebrafish, chick) may employ molecular mechanisms for stem cell specification that are not conserved in humans, a prominent example being the specification of primordial germ cells (PGCs). Germ plasm irreversibly specifies PGCs in many models; however, it is not conserved in humans, which produce PGCs from tissue termed germline-competent mesoderm (GLCM). GLCM is not conserved in organisms containing germ plasm, or even in mice, but understanding its developmental potential could unlock successful production of other stem cell types. GLCM was first discovered in embryos from the axolotl and its conservation has since been demonstrated in pigs, which develop from a flat-disc embryo like humans. Together these findings suggest that GLCM is a conserved basal trait of vertebrate embryos. Moreover, the immortal nature of germ cells suggests that immortality is retained during GLCM specification; here we suggest that the demonstrated pluripotency of GLCM accounts for retention of immortality in somatic stem cell types as well.

This article has an associated Future Leaders to Watch interview with the author of the paper.

## Abstract (precis)

**Summary:** Recent findings that germline and stem cell specification may differ between species may have important implications for regenerative medicine and the future of stem cell biology.

## Introduction

Harnessing the biology of stem cells for therapeutic intervention is a focal goal of regenerative medicine, which, in recent years, has come closer to reality. At the core of our understanding of stem cell biology lies developmental biology; decades of research have contributed to the elucidation of developmental mechanisms that regulate the natural biology of stem cells. However, recent attempts to derive stem cells, including haematopoietic (blood) stem cells (HSCs), have failed to generate *in vitro*-produced HSCs capable of long-term blood repopulation (Lis et al., 2017; Sugimura et al., 2017), suggesting that fundamental aspects of HSC development, largely acquired from studies utilising diverse model organisms, are not understood. While unifying themes regarding the development of HSCs and other stem cell types have been proposed, incomplete translation from model organisms to humans suggest another, wider-ranging, problem: that conventional model organisms cannot provide the full extent of information required to produce the same outcomes in humans. This Review will discuss the future of stem cell research employing developmental models, focussing primarily on HSCs. It will present an evolutionary argument for the use of model organisms representing basal vertebrate development, rather than those conventionally selected for expedience, since these display derived forms of development that have influenced the basis of most of our current knowledge (Bachvarova et al., 2004; Johnson et al., 2001, 2003a,b).

Research using mice, rats, chicks, frogs (*Xenopus*) and zebrafish has yielded significant advances into understanding HSC development, from *in vitro* colony-forming assays to live imaging of circulating HSCs (Kissa et al., 2008; Siminovitch et al., 1963; Wu et al., 1967). These organisms offer a wealth of attractive characteristics that enable rapid and effective research, including short generational times, external development, and genetic tractability. However, it is becoming increasingly questionable whether data from animal models established with experimental efficacy in mind, are directly translatable to development in humans, which develop slowly. For example, gene regulatory networks (GRNs) controlling mesoderm development display little consistency observed between the species listed (Brown et al., 2014). This suggests a misunderstanding of the individual species' evolution, and perhaps that the evolution of GRNs has a greater plasticity than previously thought. It is therefore important to understand how this relates to humans. The embryos of pigs, for instance, more accurately approximate human development than those of mice, and are currently gaining traction as an animal model; indeed, fundamental embryology, such as disc-shaped epiblasts, is shared between humans and pigs, whereas mice develop in a cup-shaped epiblast (Alberio et al., 2021; Johnson and Alberio, 2015).

A key developmental distinction that pigs share with humans diverges from the aforementioned model organisms: the mechanism by which the germline is specified (Kobayashi et al., 2017). This dichotomy is between induction and preformation (see Box 1 for definitions). Induction, in its most basal form, produces germ cell progenitors from germline competent mesoderm (GLCM), shortly after gastrulation. GLCM refers to a specific subset of pluripotent mesoderm cells, from which primordial germ cells (PGCs) have been shown to arise in axolotls and pigs. Importantly, while GLCM is ‘germline competent’, other mesodermal cell types may arise from it; indeed, expression of mesodermal markers not associated with germline can be identified from within the population (Chatfield et al., 2014; Kobayashi et al., 2017). PGC induction in mice, on the other hand, occurs via ‘blimping’ shortly before gastrulation (Chuva de Sousa Lopes and Roelen, 2008; Lawson et al., 1999; McLaren and Lawson, 2005), wherein Blimp1 expression defines irreversibly committed PGCs; similarly in non-mammalian models, preformation segregates the germline from the mesoderm entirely, separating presumptive germ cells from the developing soma before germ layers even form (Johnson and Alberio, 2015). While preformation has evolved convergently throughout the animal kingdom, both in vertebrates and invertebrates, this Review focusses on terrestrial and aquatic tetrapod vertebrates and seeks to identify possible interrelationships between germline and stem cell development in species that have retained the basal mode of PGC induction (basal; bPGC), which is absent in performative (derived; dPGC) species.

**Box 1. Definitions of terms used**

**bPGC:** basal mode of tetrapod PGC formation. This is typically defined as occurring through inductive signals during the process of gastrulation, often BMP signalling from somatic cells to ensure PGC lineage restriction.

**dPGC:** derived modes of PGC specification, which include use of germ plasm (via maternal mRNA deposits, for example) in multiple organisms and blimping in mice.

Stem cells and germ cells share several characteristics, importantly, including cellular immortality, or the ability to self-renew (Hiyama and Hiyama, 2007). Germline immortality is essential for the survival and continuation of species and, while germ cells and stem cells display age-related functional decline, their functionality is maintained through increased telomerase activity until organisms are sexually mature (Schultz and Sinclair, 2016; Selvaratnam et al., 2015). Two developmental possibilities therefore exist: (1) that these traits are inherited from a shared embryological origin, or (2) that they are naturally programmed into stem cells arising within somatic tissues. We postulate that development of these two lineages is linked by a common origin in the GLCM. In many developmental models, however, this link is unnecessary, as the germ line segregates prior to development of somatic tissues. This Review, therefore, aims to analyse the evolutionary differences between species regarding HSC production in relation to the mode of PGC specification with a view to highlighting potential alternative areas of study. HSCs are used as a model for the possibility that other stem cell types may follow a similar trajectory in species displaying bPGC specification. The three central possibilities this Review will highlight are:

1. that in bPGC organisms, both PGCs and other cell stem types, including HSCs, may share an origin within the GLCM. GLCM cells must maintain immortality/telomerase activity in order to maintain germline potential – traits shared with stem cells;
2. that the fundamental biology of stem cells post-specification is conserved among all species, but developmental origins differ on a species-specific basis; and
3. that many stem cell types share a naivety, which accounts for observed plasticity between cell types, meaning stem cells and PGC precursors can be reprogrammed by developmental niches, which suggests plasticity via shared traits/origins.

### The importance of mode of PGC specification in relation to development and evolution

Since bPGC specification from GLCM is the basal tetrapod mode of PGC determination, germ plasm and other dPGC modes of specification must have evolved subsequently, convergently (Johnson et al., 2003a,b). dPGC specification occurs prior to gastrulation, in all known models, and its appearance aligns with the loss of GLCM. The potential embryological benefit to dPGC specification is a liberation of mesoderm from morphological constraints necessitated by germline specification, as outlined elsewhere (Johnson and Alberio, 2015). If so, wider ranges of morphology are possible, and, indeed, are observed in dPGC species. Furthermore, human PGC-like cells (PGCLCs), are most-successfully induced via a mesoderm-like state (Sasaki et al., 2015), suggesting a mesoderm (and therefore possible GLCM) origin in humans. To definitively model early human PGC development, understanding PGC development in bPGC species is essential. That is not to say that broad regulation, post-specification and lineage restriction, may not be conserved between induction and preformation.

Preformation is distributed throughout the animal kingdom. In zebrafish, maternally deposited factors in the animal hemisphere regulate the specification of PGCs and are asymmetrically distributed during early developmental cleavages (Braat et al., 1999; Hartung et al., 2014; Knaut et al., 2000; Olsen et al., 1997; Weidinger et al., 2003; Yoon et al., 1997). *Xenopus* PGCs, conversely inherit germ plasm determinants within the vegetal cortex of the egg (Houston and King, 2000), and PGC initiation is induced via repression of transcription and translation of zygotic gene expression (Lai et al., 2012; Venkatarama et al., 2010), preventing responses to somatic signals. In these two species, therefore, PGCs are the first cell lineage to be specified in development.

Mouse PGC specification is initiated around E6.5 (prior to gastrulation), via inductive signals, which is earlier than in other mammals. In mice, BMP4 signalling from the extra-embryonic ectoderm induces WNT3a, which in turn activates T. T is responsible for Blimp1 expression (‘blimping’; Aramaki et al., 2013; Lawson et al., 1999; McLaren and Lawson, 2005; Surani et al., 2004) during mouse PGC specification. Concurrently, specified PGCs downregulate somatic genes, including hox genes, by mid-late streak stages (E6.5–E7.0) (Kurimoto et al., 2008; Ohinata et al., 2005). Conversely, PGC- and pluripotency-associated genes (Stella, Sox2, Nanog) are either maintained within the population, or display reduction followed by recovery (Kurimoto et al., 2008; Yamaguchi et al., 2005). Early mouse PGCs are observed by E7.0, reaching the genital ridges by E10.5 (Molyneaux and Wylie, 2004; Richardson and Lehmann, 2010).

Although mouse PGCs are not specified by maternal deposits, PGC specification occurs prior to gastrulation, which is also the case during specification via germ plasm. Importantly, a subset of cells is predetermined as PGCs, via transcriptional repression of somatic genes and maintenance of pluripotency factors, prior to the definitive determination of germ layers. Yet, despite the conservation of inductive signals that give rise to PGCs in mammals, the tissue of origin in mice is not definitively mesodermal, in contrast to other mammals. Mouse PGC specification therefore represents a derived (dPGC) form of PGC specification in relation to other mammalian species. Indeed, the basal rodent, *Lagostomus maximus*, induces PGCs during gastrulation and displays a disc-shaped epiblast, indicating blimping evolved within the rodent lineage (Leopardo and Vitullo, 2017); *L. maximus* PGC specification, characterized by expression of *SOX17* and *BLIMP1*, is therefore more similar to humans and pigs (and other non-rodent mammals), further highlighting how mouse PGC specification and embryology has evolved to differ from most other mammals.

**Fig. 1.**
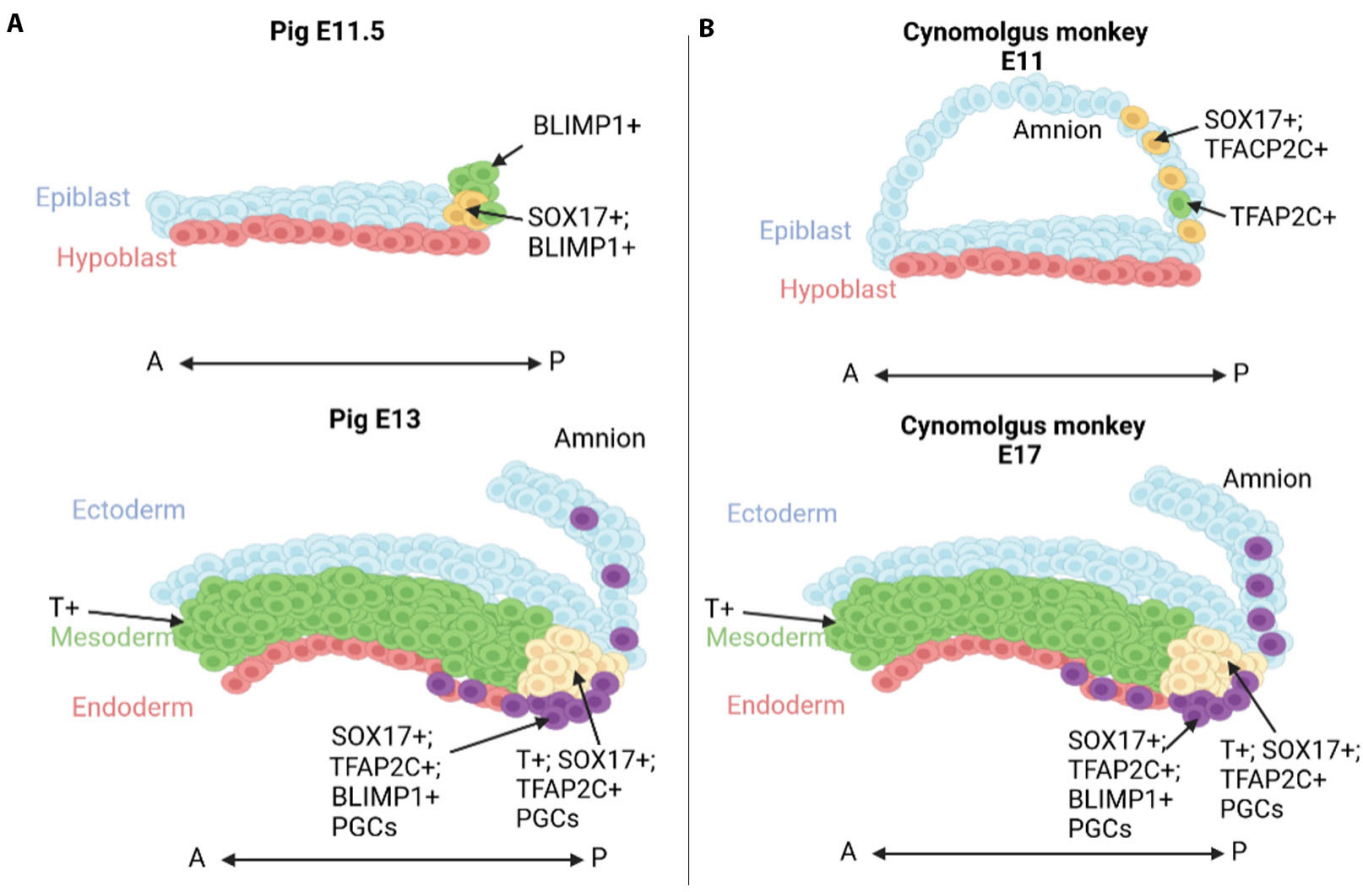
**Specification of PGCs in non-rodent mammals and primates display similarities during gastrulation despite differential PGC markers during earlier development.** (A) Porcine embryos form an amnion after implantation, while (B) primate embryos (illustrated by cynomolgus monkey) form an amnion before implantation. SOX17/BLIMP1+ cells are observed in the porcine posterior epiblast prior to gastrulation, while similar cells are observed in the amnion of primates. However, during gastrulation in both species, increased numbers of PGCs are observed in the posterior epiblast. At E13 pig and E17 cynomolgus monkey, yellow cells indicate T+; SOX17+; TFAP2C+ PGCs induced in GLCM, purple cells indicate migratory SOX17+; TFAP2C+; BLIMP1+ PGCs. Image created in Biorender.

Human and porcine PGCs (along with most other bPGC species), on the other hand, are specified following onset of gastrulation, from *EOMES*- and *T*-expressing precursors (Kojima et al., 2017; Chen et al., 2017). Upon BMP4 induction *SOX17* triggers development of the PGC developmental program and *BLIMP1* represses somatic lineage potential (Irie et al., 2015; Kobayashi et al., 2017). Porcine PGCs emerge in the posterior epiblast during gastrulation and can be identified by expression of pluripotency factors (*OCT4*, *NANOG*) and *SOX17*, which is also observed prior to *BLIMP1* (Fig. 1A). Importantly, pig PGC induction occurs within a subset of cells expressing mesodermal genes, are induced by hypoblast cells which express *BMP2* and *WNT* signalling and activate PGC fate via induction of *BMP4* expression (Johnson and Alberio, 2015; Kobayashi and Surani, 2018; Kobayashi et al., 2017). GLCM has been studied in non-mammalian species as well and was first characterised in the embryos of axolotls (Chatfield et al., 2014; Nieuwkoop, 1947; Sutasurja and Nieuwkoop, 1974). Axolotl PGCs are derived from GLCM cells in the ventral marginal zone *in vivo*, which can also give rise to somatic cells, and specification also occurs after gastrulation. Furthermore, perturbations to PGC specification even after gastrulation induce a mesodermal fate at the expense of the germline (Chatfield et al., 2014), thus indicating the relatively late process of germ line restriction.

Interestingly, primate PGC specification appears to occur in amnion cells prior to gastrulation (Sasaki et al., 2016) (Fig. 1B). The primate amnion forms from epiblast cells prior to implantation and maintains expression of OCT-4, NANOG and SOX2, suggesting that the early amnion remains competent to respond to PGC inducing molecules. Importantly PGCs do not remain in the amnion, instead migrating to the posterior epiblast during gastrulation (Sasaki et al., 2016). In this way, it is possible that while primate PGC formation displays some divergence from basal mammal specification, the cells which are observed in the amnion may indicate a broader role for GLCM in primates (Fig. 1). In the pig, where the formation of the amnion is delayed until after the onset of gastrulation, the PGCs are first found in the posterior epiblast and the population expands for several days at the same time as they start their migration towards the gonad. Notably, during this time migratory cells can also be found in ectopic locations such as the amnion. Future investigations on PGC origin in other mammals are needed to help establish whether the amnion origin of the germline is an evolutionary novelty of certain primates.

The axolotl models development of the basal terrestrial vertebrate body plan, resembling the first tetrapods to colonise land, with fossil records spanning over 300 million years (Johnson et al., 2003a,b). Conversely, frogs, used as models for understanding vertebrate development for decades, have undergone significant deviations from the basal body plan, through around 275 million years of evolution (Zhang et al., 2013). In this regard, it has been proposed that precocious specification of PGCs by germ plasm, or even blimping, liberates genetic constraints on development that enable deviation from the basal vertebrate body plan. Moreover, the appearance of precocious PGC specification aligns with the loss of GLCM, meaning the fate of GLCM cells that do not become PGCs cannot be explored in dPGC organisms (Johnson and Alberio, 2015). Therefore, studying GLCM in bPGC species is essential to understanding human development. Since not all cells in the GLCM become PGCs, the eventual fate of those cells is an important aspect of human development that is currently unexplored.

Cultured human PGC-like cells which lack *BLIMP1* display expression of mesodermal markers including *KDR* (endothelial), *gGATA2* (blood), *PDGFRA* and *hHAND1* (cardiac) (Tang et al., 2015), suggesting other mesodermal lineages may be derived from mesodermal cells that do not receive signals to form PGCs and that PGC fate must be conferred by extracellular effectors, non-cell autonomously.

**Fig. 2.**
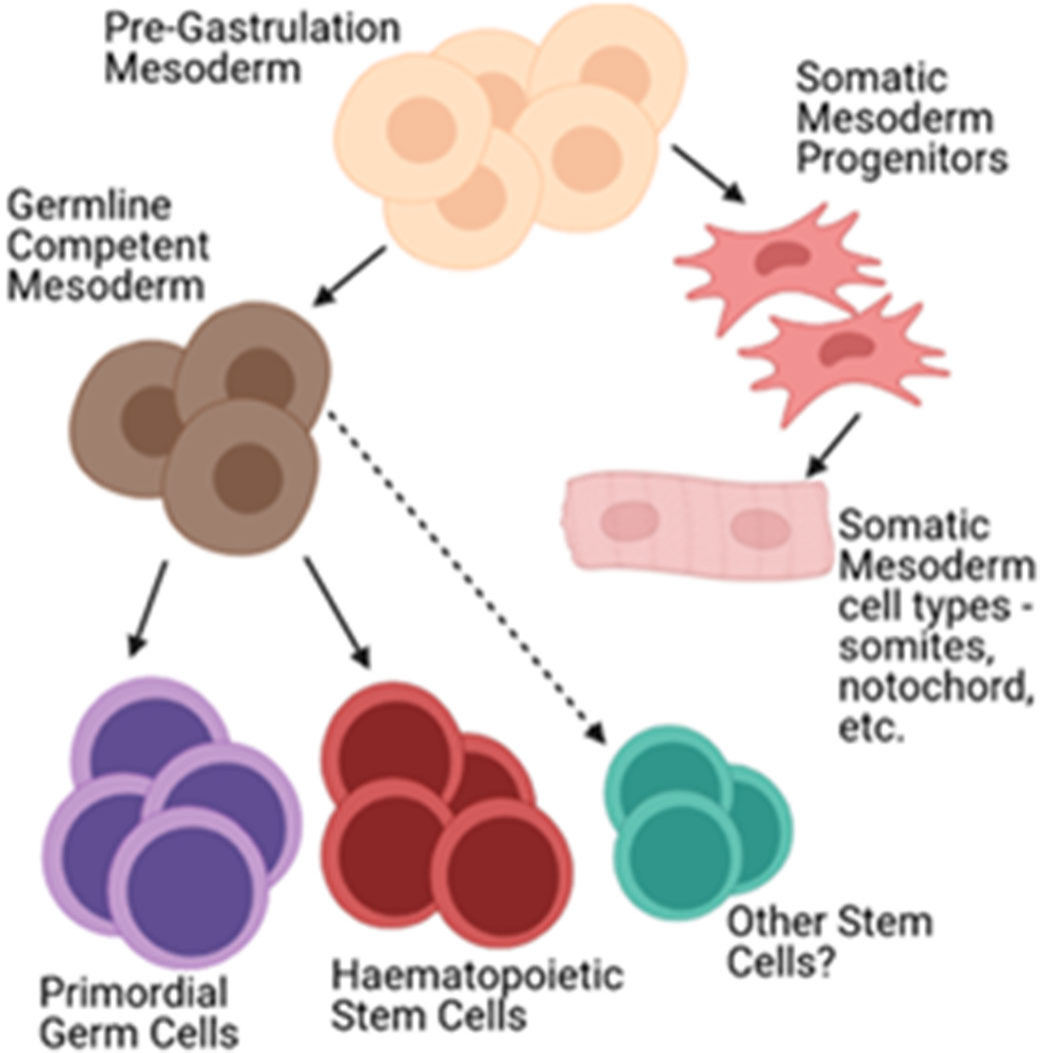
**Potential stem cell differentiation hierarchy in bPGC species.** In species which specify PGCs via GLCM, different stem cell types (including HSCs) may also be specified simultaneously and therefore directly share ancestry with PGCs. Image created in Biorender.

Stem cells retain key characteristics from GLCM (immortality, self-renewal and multipotency). Therefore, it seems possible that GLCM mesoderm ensures PGCs are specified prior to any other cell type, while allowing other cells derived from GLCM to maintain pluripotency-associated characteristics. This may represent a ‘blank slate’ stem cell type that can be influenced by somatic cells to become a resident stem cell. Interestingly, transience of stem cell status has been suggested, which may provide stem cells with a plasticity prior to specification (Zipori, 2004). Therefore, it seems possible that cell populations either inherit ‘stemness’ from the GLCM, and continue to function as stem cells, or do not, and instead give rise to somatic tissues, including those responsible for maintaining the niches that support stem cell growth and differentiation. It is likely that this ‘stemness’ (or ground state) might include expression of OCT4, NANOG and SOX2, marking pluripotency, along with mesodermal markers, including KDR and PDGFRA (Tang et al., 2015). The question remains as to whether cells which arise within GLCM display pre-determined cell lineage restrictions, or whether a degree of stochasticity regulates pluripotent precursors cells in response to signalling gradients released from niches. Therefore, we postulate that the GLCM is essential for not only PGC formation, but also other somatic stem cell populations (Fig. 2).

### Differential regulation of haematopoietic stem cell developmental biology in different species and their similarity to PGCs

The suggestion that PGCs and HSCs display similarities or are related developmentally is not new, conceptually (Rich, 1995), but recent evidence strengthens the argument that after gastrulation a subset of mesodermal cells retain germ line competence and multipotency prior to cell-specialization.

Recent studies have come increasingly close to generating HSCs *in vitro*, through the use of elegant culture environments supplemented with specific transcription factors (including *RUNX1*, *GFI1*, *HOX9A* and *ERG*), which direct the formation of HSC-like cells from either haemogenic endothelial cells, or induced pluripotent stem cells (Lis et al., 2017; Sugimura et al., 2017). dPGC animal models were instrumental in determining functions of essential haematopoietic transcription factors. It is therefore important to discuss discoveries made using dPGC organisms to understand the similarities and probable differences between HSC specification compared to bPGC species.

HSCs were initially identified using mouse bone marrow transplantation into irradiated donors (Becker et al., 1963; Till and McCulloch, 1961; Wu et al., 1967) and embryological analyses discovered that haematopoiesis occurs in multiple waves (Ciau-Uitz et al., 2000; Detrich et al., 1995; Huber et al., 2004; Lopez et al., 2014; Padrón-Barthe et al., 2014; Palis and Kingsley, 1995; Palis et al., 1999; Silver and Palis, 1997). Definitive HSCs arise primarily in the haemogenic endothelium of the dorsal aorta (DA) (de Bruijn, 2000) before migrating to and maturing in sequential niches. In mice, intra-aortic haematopoietic clusters (IAHCs) emerge primarily from the ventral wall of the DA (Bertrand et al., 2010; De Bruijn et al., 2002; Kissa and Herbomel, 2010), although dorsal wall HSC emergence has been observed (Taoudi and Medvinsky, 2007; Taylor et al., 2010a). HSCs later colonise the foetal liver and undergo expansion (Ema and Nakauchi, 2000) before migration to the bone marrow, forming a niche with mesenchymal stem cells (MSCs) (Méndez-Ferrer et al., 2010). In zebrafish, HSCs emerge in the dorsal aorta and migrate to the caudal haematopoietic tissue (analogous to the foetal liver) (Murayama et al., 2006), maturing before migration to the thymus and kidney marrow (Murayama et al., 2006). Thus, in both mice and zebrafish, definitive HSCs first emerge in (or around) the DA, before migrating through successive tissues during maturation. Identification of the progenitors from which they originate, however, is less clear.

Definitive haematopoiesis is regulated by a cascade of transcription factors that were principally characterised using model organisms. Moreover, an impressive map of the gene regulatory network for HSC specification has developed. HSC regulation by *runx1*, *c-myb*, *scl/Tal1*, *BMP-4 and gata-2* has been established (Bussmann et al., 2007; Butko et al., 2015; de Pater et al., 2013; Gering et al., 1998; Qian et al., 2007; Reischauer et al., 2016; Rybtsov et al., 2014; Soza-Ried et al., 2010; Stainier et al., 1995; Thompson et al., 1998; Wilkinson et al., 2009) through model organism-driven embryological research. Indeed, the relevance of such findings has been highlighted by their translation to human cell lines; *RUNX1* and *SCL/TAL1* regulate human HSC self-renewal (Gerritsen et al., 2019; Reynaud et al., 2005), while *BMP4* and *c-MYB* regulate HSC differentiation (Jeong et al., 2020; Shah et al., 2021) and *GATA2* regulates HSC cell cycle entry (Tipping et al., 2009). Importantly, however, the majority of this understanding is related to HSC specification which occurs relatively late in development; characterisation of early HSC development prior to their emergence within the dorsal aorta is less well understood, and, indeed, likely cannot be fully understood using dPGC species alone.

Ablation of transcription factor function impairs haematopoiesis; *runx1* deficiency in both mice and zebrafish impairs blood formation and reduces emergence of definitive HSCs from the aortic endothelium (Burns et al., 2005; Chen et al., 2009; Growney et al., 2005; Kalev-Zylinska et al., 2002; North et al., 1999, 2002; Okuda et al., 1996; Wang et al., 1996); a requirement for *Gfi1b* during mouse erythropoiesis and megakaryopoiesis has been established (Saleque et al., 2002) and *gfi1aa/gfi1b* mutant zebrafish display erythropoiesis deficiencies (Moore et al., 2018; Thambyrajah et al., 2016), while *erg* maintains HSC self-renewal in mice (Knudsen et al., 2015). Moreover, *hoxa9* abrogation causes defective erythroid, myeloid and lymphoid haematopoiesis, reducing repopulating capabilities of HSCS in mice and causing anaemia in zebrafish (Deveau et al., 2015; Lawrence et al., 1997, 2005). *Hoxa10* overexpression impairs myeloid differentiation in human haematopoietic progenitors (Buske et al., 2001) and aberrant *hoxa5* expression drives accumulation of megakaryocyte-erythroid progenitor cells in mice (Yang et al., 2015). The genes listed were used to produce HSC-like cells *in vitro* in recent studies, wherein HSC-like cells were produced via a haemogenic endothelium intermediate (Lis et al., 2017; Sugimura et al., 2017), highlighting the importance of model organism-based studies.

Importantly, human embryos also display haemogenic endothelium (Oberlin et al., 2002) and CD34+/CD45+ IAHCs associate with the ventral wall of the dorsal aorta in human embryos (Tavian et al., 1996, 1999), suggesting strongly that late aspects of haematopoiesis are conserved regardless of origin. Moreover, angiotensin converting enzyme (ACE) expression marks human haematopoietic cells (Jokubaitis et al., 2008; Sinka et al., 2012); in contrast with observations from model organisms, however, KDR+/ACE+ cells appear to migrate towards the dorsal aorta instead of arising from it, suggesting the origins of haemogenic-competent endothelial cells may differ between species, while later regulation may remain similar.

It is possible that migrating KDR+/ACE+ cells could represent immature haemogenic endothelium or HSC precursors (Jokubaitis et al., 2008; Sinka et al., 2012). Human embryos also display discontinuous DA endothelium during the formation of IAHCs and cells appear to migrate from the sub-aortic mesenchyme into the aortic lumen (Tavian et al., 1999). Intriguingly, migratory ACE-labelled cells also express pluripotency markers (OCT-4, SSEA1) during migration, markers that are also expressed by simultaneously migrating PGCs (Jokubaitis et al., 2008; Sinka et al., 2012). Moreover, the DA already exists prior to this migratory event, suggesting a distinction between HSCs and endothelial cells in humans. This suggests human HSCs can contribute to endothelial structures that already exist, similar to observations in mice (Ganuza et al., 2018; Plein et al., 2018; Stefanska et al., 2017). What is not clear in human embryos, however, is whether the endothelium of humans is competent to form HSCs prior to the migration of these cells. Furthermore, debate exists as to whether HSCs always arise initially in the DA endothelium in model organisms; evidence for HSCs in the subaortic space in mice and zebrafish during at least part of their development has also been observed (Bertrand et al., 2005; Kissa and Herbomel, 2010), while *Xenopus* embryos also display *scl* expression ventral to the dorsal aorta prior to IAHC emergence (Ciau-Uitz et al., 2000). Furthermore, BMP-ER, a regulator of HSC maturation, is expressed extensively throughout the subaortic mesenchyme during HSC development in mice (McGarvey et al., 2017).

Taken together, these studies indicate a need to reassess early HSC formation, prior DA emergence, to glean insight into conserved mechanisms of stem cell development in vertebrates. It may be possible that partial maturation within the subaortic mesenchyme is essential and conserved throughout species. However, dPGC specification obviates GLCM requirements (Johnson and Alberio, 2015) and severs any possible developmental connection between PGCs and HSCs. Evolution of dPGC mechanisms might therefore coincide novel HSC specification. Indeed, *cloche* (*npas4l*) is known as a master regulator of HSC and endothelial development in zebrafish but is not present in mammalian genomes (Reischauer et al., 2016; Stainier et al., 1995). The mechanism by which *cloche* specifies the HSC lineage in zebrafish is still not entirely clear, although it may function during the induction of HSC- and endothelial-specific genes (Marass et al., 2019).

**Fig. 3.**
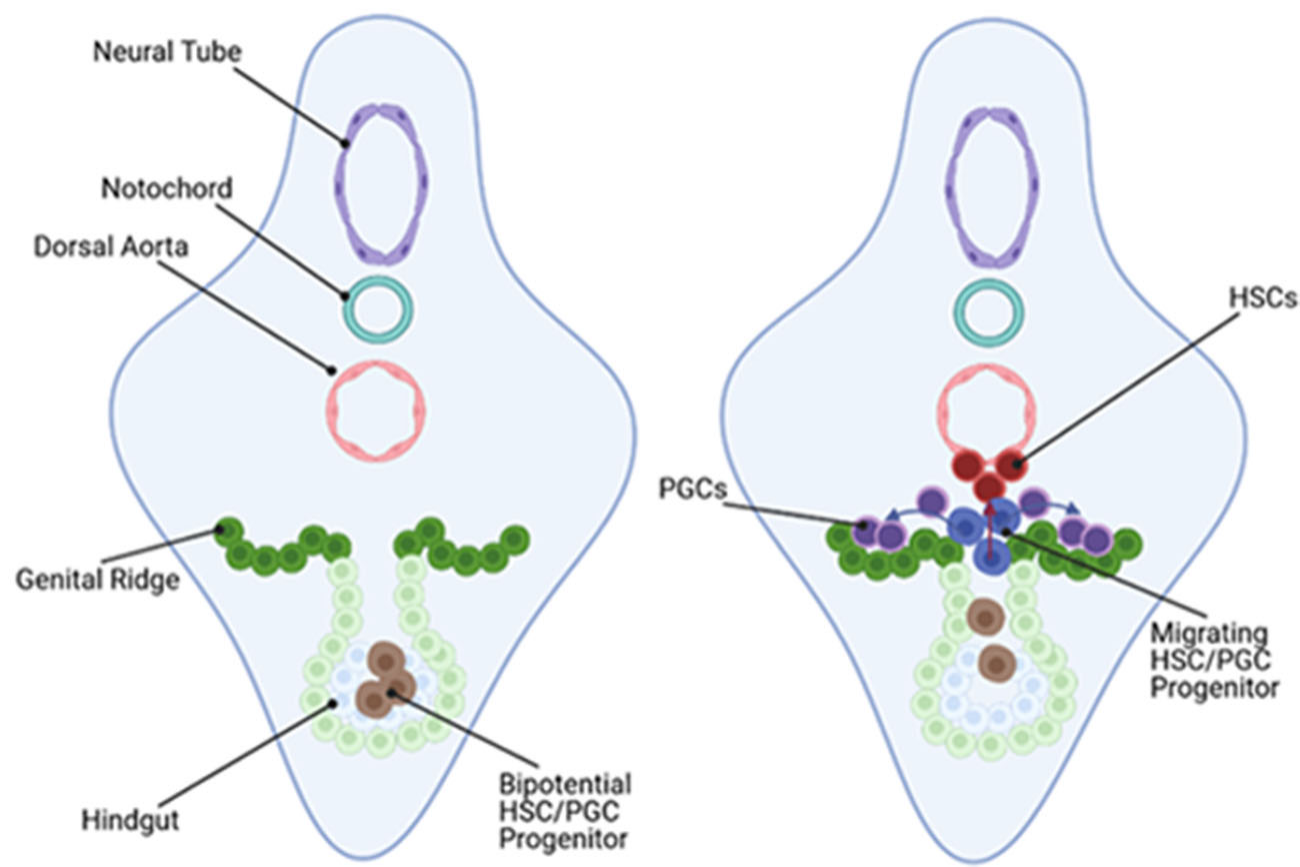
**Predicted basal vertebrate migratory route for PGCs and HSCs.** In Carnegie stage 10 (∼21 days in human embryos) bPGC embryos, pre-migratory bipotential progenitors are associated with the hindgut; Carnegie stage 12 (∼27 days in human embryos) bPGC embryos display migratory cells expressing both PGC and HSC markers. These cells colonise both the genital ridges and ventral wall of the DA, suggesting they respond stochastically to migratory cues from each tissue. Image created in Biorender.

Therefore, though human HSC development shares similarities with those of dPGC organisms, species-specific divergences indicate that, like PGCs, the entirety of HSC specification is not conserved amongst all species. It is important to note that, in human embryos, presumptive migratory HSCs display overlapping expression profiles with PGCs that migrate to the AGM concurrently (Sinka et al., 2012). We therefore suggest a potential, evolutionarily basal, mode of tetrapod PGC/HSC specification, wherein a bipotential progenitor emerges within the GLCM, which migrates towards the AGM via the hindgut (Fig. 3). We predict that a stochastic response to migratory cues determines whether bipotential progenitors migrate to the DA or genital ridges (Fig. 3B). However, lineage tracing experiments would be necessary to determine this, such as making use of the CAGGs: LP-EGFP-LP-Cherry axolotl line (Khattak et al., 2013) in combination with Cre recombinase expression driven by the axolotl DAZL promoter, to switch GFP expression to Cherry in all descendent cells. Since axolotls develop externally, outside of a uterus, this can be analysed more efficiently than in mammalian embryos. These experiments could provide precedent for equivalent experiments in embryos from non-rodent mammals; interestingly, dual fluorochrome pig embryos have been generated using the ROSA26 locus, enabling fluorophore switching from RFP to GFP in cells exposed to Cre recombinase activity (Li et al., 2014). Generation of embryonic stem cell lines from which gastruloids can be produced would enable powerful *ex vivo* developmental studies in non-rodent mammals. For example, CRISPR knock-in of Cre downstream of the DAZL promoter would enable lineage tracing in pig gastruloids throughout early development.

### Similarity between stem cell types and plasticity in differentiation

**Fig. 4.**
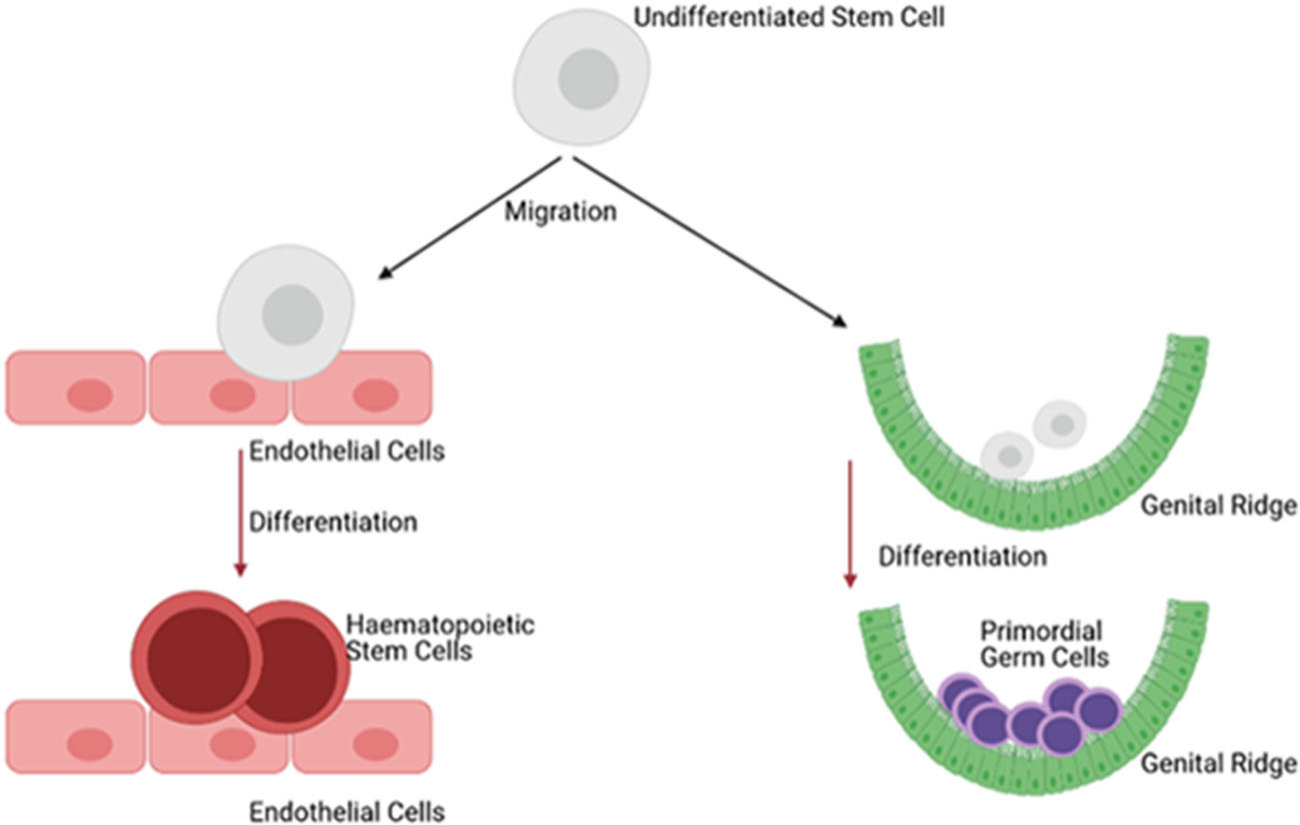
**Engraftment into different niches can confer a new identify on stem cells.** Studies have shown that stem cells are capable of producing cell types relevant to the somatic tissue onto which they are engrafted, suggesting a plasticity of stem cell nature. Image created in Biorender.

In this Review, we suggest PGCs and HSCs, and likely other stem cells, are all derived from a common precursor pool within the GLCM of many species, including humans. PGCs prior to the completion of blimping also show a potential to change fate when engrafted into different developmental niches (Rich, 1995). Therefore, it is possible that supposedly lineage-restricted stem cells develop from a more fundamental state of ‘stemness’, which is relatively plastic; in fact, it is possible that the surrounding niche (comprised of somatic cells) directly regulates gene expression to ensure the plastic stem cell, or PGC, undergoes restriction to produce the necessary somatic cell types for normal tissue function (Fig. 4).

Human presumptive HSCs express PGC markers (Oct-4, SSEA) during migration (Jokubaitis et al., 2008; Sinka et al., 2012) and BMP-ER, an endothelially-expressed HSC regulator, is also expressed in cells that contact PGCs during their development in mice (McGarvey et al., 2017). Furthermore, PGCs have been shown to display haematopoietic/endothelial activity (Scaldaferri et al., 2015). This indicates some overlap in transcriptional phenotype of both HSCs and PGCs. Moreover, PGCs and HSCs arise in close proximity (within the AGM). Indeed, in axolotl embryos, GLCM cells co-express *axdazl* and *axscl* (Bachvarova et al., 2004), suggesting the existence of cells which can give rise to both PGCs and HSCs. That this is found in axolotls also implies it is an ancient trait of terrestrial vertebrates, so any variation from this represents an evolutionary novelty.

At another level, very small embryonic-like stem cells (VSELs) have been shown to reside in multiple adult tissues, including adult mouse bone marrow (Kucia et al., 2006, 2008). They express germ cell markers including SSEA-1 (Kucia et al., 2006), alongside markers for both HSCs (CD45) (Kucia et al., 2006) and neural stem cells (Nestin) (Shaikh et al., 2017), suggesting they may be derived from pluripotent mesoderm precursors and function as undifferentiated banks for more specialised stem cell types. Moreover, PGCs have been isolated from CD31+ human embryonic cells alongside endothelial and haematopoietic cells (Crosse et al., 2020). CD31 is typically identified as an endothelial marker, so the identification of cells with a PGC expression profile, though acting as an outlier, is unexpected, unless both cell types are derived from a common precursor pool before diverging.

Interestingly, both PGCs and HSCs follow a similar maturation profile, within their respective niches and both cells retain immortality. However, an unresolved question remains as to whether the immortality of HSCs is inherited or induced. Both PGCs and HSCs begin in undifferentiated ground-states, before becoming lineage primed and, during this transition, commonalities between expression profiles of both cell types have been observed, including the expression of CEBP genes (Chen et al., 2020; Fidanza et al., 2020). It is possible that the earliest ground-state cells are from the same pool of progenitors but external influences from the somatic cells of successively encountered niches suppresses plasticity, restricting precursors to the relevant lineage, and this may be reversible. The time point at which lineage restriction occurs, however, is not clear. Furthermore, cells obtained from the urogenital region of the human embryonic AGM can impart long-term engrafting HSCs to mouse embryos (Easterbrook et al., 2019). This region would contain presumptive PGCs but clearly must also include HSCs (Medvinsky and Dzierzak, 1996). Importantly, the urogenital region showed similar engraftment potential to the DA, the site of definitive haematopoiesis, alone, indicating a similar potential to form HSCs.

Many examples exist in which stem cell transplantation into different tissues or niches has resulted in modification of stem cell-mediated somatic cell production to a fate aligned with the tissues into which they are transplanted. This further suggests a degree of plasticity between stem cell types, and that a combination of a stemness fate and the niche in which it resides may be key factors for stem cell identity, including PGCs. Human HSCs have been differentiated into motor neuron-like cells *in vitro* (Moghaddam et al., 2017) and peripheral blood-derived HSCs and mononuclear cells can be converted into neural-like cells in culture with primary rat neurons (Kodama et al., 2006; Tanabe et al., 2018). Furthermore, iPSCs induced from peripheral blood cells differentiate into neural cells using neural differentiation media (Nagai et al., 2018; Tsai et al., 2015). Moreover, PGC progenitors can be directed towards somatic mesodermal fate in *ex vivo* cultures (Chatfield et al., 2014).

Interestingly, PGCs have been shown to have the potential to form HSCs in culture; mouse PGCs can form haematopoietic cells, though potential reduces as blimping takes place (Rich, 1995; Surani et al., 2004) and pluripotent stem cells derived from neonatal mouse testis/spermatogonia were capable of generating multiple cell lineages *in vitro*, including haematopoietic/endothelial cells (Kanatsu-Shinohara et al., 2004, 2008). These studies highlight the potential for stem cells to produce somatic cells unrelated to their assumed cell type. Indeed, if one considers the precursors of PGCs as a form of immortal germline/somatic stem cell progenitor, then PGCs can be included in this analysis.

Clearly, similarities (and interconvertibility) between stem cells and PGCs indicate conservation of important properties, likely: immortality and self-renewal, and multipotency in the cell types they can give rise to. From basal tetrapod embryology, it is also likely that the conserved properties are inherited from the same origin – the GLCM (Chatfield et al., 2014) – indicating the need for a deeper developmental understanding of how stem cells and PGCs form initially, during embryogenesis. Since humans likely produce PGCs via GLCM, understanding this process will have significant clinical and therapeutic benefit.

### Conclusions and future prospects for stem cell developmental biology

Huge strides have been made in understanding the development of stem cells, with dPGC model organisms being integral to the progress. However, the abundance of useful information gained from such organisms may have biased our understanding towards developmental mechanisms evolved anew by dPGC species. Therefore, our understanding of human stem cell origins, may have been impeded. Indeed, in the absence of GLCM perhaps novel mechanisms evolved in such species to expedite a more efficient specification of stem cells. Indeed, findings from model organisms have not translated well to the clinical production of human HSCs *in vitro*, highlighting fundamental differences between stem cell specification in humans and model dPGC species. Furthermore, studies using human embryos have suggested that the developmental origins of HSCs in humans may not resemble those of organisms such as mice and zebrafish (Jokubaitis et al., 2008; Sinka et al., 2012; Tavian et al., 1999). This may mean that mechanisms essential to the specification of HSCs are not conserved between dPGC species and bPGC species (like humans).

This leads to the core concept of this Review: model organisms that retain basal vertebrate embryology, including humans, pigs and axolotl, may differ significantly during the specification of stem cells compared to dPGC model organisms. Moreover, data obtained from dPGC specification may have not only clouded medically-relevant understanding of PGC formation but also understanding of the embryological origins of stem cells, reducing effectiveness in producing stem cells *in vitro*. Therefore, to improve both understanding and applications for regenerative medicine, it is essential that studies into organisms that retain bPGC specification are undertaken.

HSCs and PGCs have been observed to follow similar migratory paths across the AGM in humans, expressing some markers in common, and other distinct markers (Jokubaitis et al., 2008; Sinka et al., 2012). Moreover, pluripotency and immortality are maintained within GLCM cells (Irie et al., 2015; Johnson and Alberio, 2015) – indeed this is essential since the population contains germline progenitors; both characteristics are essential for the survival and function of germ cells, HSCs, and other stem cells. However, during development, cell types become ever more restricted, and it is likely that this is demonstrated through decreasing inheritance of the properties inherent to GLCM (pluripotency and immortality); stem cells display diminished multipotency while maintaining immortality. This is likely to be true in humans but is a hypothesis that cannot be addressed through the use of species which specify PGCs via preformation due to large-scale divergences in mesoderm development. Therefore, in species that retain basal vertebrate embryology, GLCM may well function as a source of stem cells, providing mesodermally-derived tissues (and potentially non-mesodermal tissues) with the cells required for long-term, adult tissue maintenance. This may originate via a ‘blank slate’ stem cell, ready to be induced to the requisite cell type within a tissue (Fig. 4). This follows suggestions from studies that have shown transdifferentiation of stem cells in co-culture with somatic tissues that differ significantly from their tissue of origin and suggests that, rather than stem cells being developmentally pre-determined, somatic cells are the driver of stem cell fate, inducing the formation of a source of cells relevant to the surrounding tissues.

Most importantly, a greater understanding of the developmental biology of GLCM may provide a framework for future stem cell and regenerative medicine studies, with the developmental biology of humans in mind. Indeed, many tissue types targeted by regenerative medicine studies are of mesodermal origin, including blood and endothelium, bone, and muscle cells; elucidating the gene regulatory networks required to generate progenitors *in vitro* and to allow differentiation into specified cell types will improve future therapies for a range of diseases. In this Review we have attempted to highlight the importance of understanding the evolutionary history of a cell type, in this case the development of HSCs, as a means of developing methods to maximize their use for therapeutic purposes. GLCM is an ancient vertebrate trait that is not conserved in conventional animal models and understanding its full developmental potential could lead to stem cell-based therapies currently beyond our reach due to limitations of *in vitro* stem cell induction.
